# Acronyms, Shmacronyms

**DOI:** 10.1100/tsw.2001.12

**Published:** 2001-03-23

## Abstract

Nature leads off this week with a story about a German proposal to build new collider for high-energy physicists named TESLA. Science leads with an article about a Soviet proposal to convert a former bioweapons lab called VECTOR into a center for virus research.

Officials at Russia's State Research Center of Virology and Biotechnology, which goes by the undecipherable acronym of VECTOR, are trying to convince everyone from CNN-mogul-turned-nonproliferation-angel Ted Turner to the World Health Organization that they deserve $25 million dollars up front and a $12 billion a year allowance. The money would be used to convert VECTOR, the only biosafety level 4 lab in Asia, from weapons research to an international center for emerging diseases, reports *Science*. The new lab would be called the International Center for the Study of Emerging and Reemerging Diseases (ICERID?). Supporters of the plan note that VECTOR scientists work with some of the world's deadliest viruses, including smallpox, tick-borne encephalitis, and hemorrhagic fever viruses, reports *Science*. But others aren't so sure it is a good idea. An unidentified virologist told *Science* that VECTOR scientists are too focused on bioweapons threats and have been slow to embrace hot topics like foot-and-mouth disease. "They do not think outside the box," contends the mystery guest. Russian officials will make their first pitch for ICERID at the Ted-Turner-funded Sam Nunn Policy Forum to be held next week at Georgia Tech University in Atlanta, Georgia.

This week an international team of high-energy physicists will argue that the future of their discipline lies, at least partly, in Germany, reports *Nature* in their lead story. The machine they want to build is called TESLA, an acronym that is creatively derived from "Tera electron volt Energy Superconducting Linear Accelerator". (TEVESCLA just didn't sound as good.) TESLA would be capable of colliding pairs of electrons and positrons at energies of more than 500 giga-electron volts, reports *Nature*. The announcement attracted widespread attention because the U.S. and Japan both have made comparable proposals to build a linear collider in their countries. Although scientists are downplaying any competition between the countries, says *Nature*, a sizable U.S.-Japanese contribution is already helping build the Large Hadron Collider in Geneva. Putting TESLA in Germany would "mark the virtual eclipse of particle physics in the United States and Japan for a generation," says *Nature*. The issue will surely be discussed when U.S. particle physicists meet in Snowmass, Colorado this summer to set future priorities for the field. But the final decision is likely to be made by the respective governments, reports *Nature*. Neither Germany, Japan, nor the U.S. is willing to foot the entire bill for a new collider.

Biologists are threatening mutiny if the International Space Station (ISS) research budget is slashed by $4 billion as proposed by the Bush administration, report both *Science* and *Nature*. The scientific mission of ISS has always had its doubters in the science community, report the journals, but this time it is the station's staunchest supporters who are threatening to walk out. NASA officials advised patience.
